# Wnt signalling in lung development and diseases

**DOI:** 10.1186/1465-9921-7-15

**Published:** 2006-01-26

**Authors:** Judit E Pongracz, Robert A Stockley

**Affiliations:** 1 Department of Immunology and Biotechnology, University of Pécs, Pécs, Hungary; 2 Institute for Biomedical Research, University of Birmingham, Birmingham, UK; 3 Department of Medicine, University of Birmingham, Birmingham, UK

## Abstract

There are several signalling pathways involved in lung organogenesis including Notch, TGFβ /BMP, Sonic hedgehog (Shh), FGF, EGF, and Wnt. Despite the widely acknowledged significance of Wnt signalling in embryonic lung development, the role of different Wnt pathways in lung pathologies has been slow to emerge.

In this review, we will present a synopsis of current Wnt research with particular attention paid to the role of Wnt signals in lung development and in pulmonary diseases.

## Overview of Wnt signalling

The Wnt family of 19 secreted glycoproteins control a variety of developmental processes including cell fate specification, proliferation, polarity and migration. Consequently, mis-regulation of Wnt signalling during embryonic development cause developmental defects, while defective Wnt signalling in adult tissue results in the development of various diseases [1]. As Wnt-s have a diverse role in regulating cell functions, Wnt signalling is predictably complex. Wnt family members bind to cell surface receptors called Frizzleds (Fz) and trigger intracellular signalling cascades. The 10 Fz proteins are members of the seven-loop transmembrane receptor family, and are encoded by 9 genes. The assembly of an active receptor complex also requires the presence of the co-receptor low density lipoprotein related protein (LRP) 5/6.

There are at least three signalling pathways involved in the signal transduction process: the canonical or β-catenin dependent, and two non-canonical: the polar cell polarity (PCP) or c-Jun N-terminal kinase (JNK)/ activating protein (AP) 1 dependent and the Ca2+ or protein kinase C (PKC)/Calmodulin kinase (CaMK) II/ nuclear factor of activated T cells (NFAT) dependent signalling pathways. Wnt signalling is modulated by numerous regulatory molecules (for a review see [1,2]) and by frequent interactions amongst the pathways themselves [3]. Wnt molecules have been grouped as canonical (Wnt1, Wnt3, Wnt3a, Wnt7a, Wnt7b, Wnt8) and non-canonical pathway activators (Wnt5a, Wnt4, Wnt11) [4]. The ability of the two groups to trigger canonical or non-canonical signalling cascades, however, is not absolute. Promiscuity of Wnt-s and their receptors are a feature of this developmentally and pathologically important glycoprotein family making studies of Wnt signalling difficult.

### Canonical Wnt-pathway

**Figure 1:**
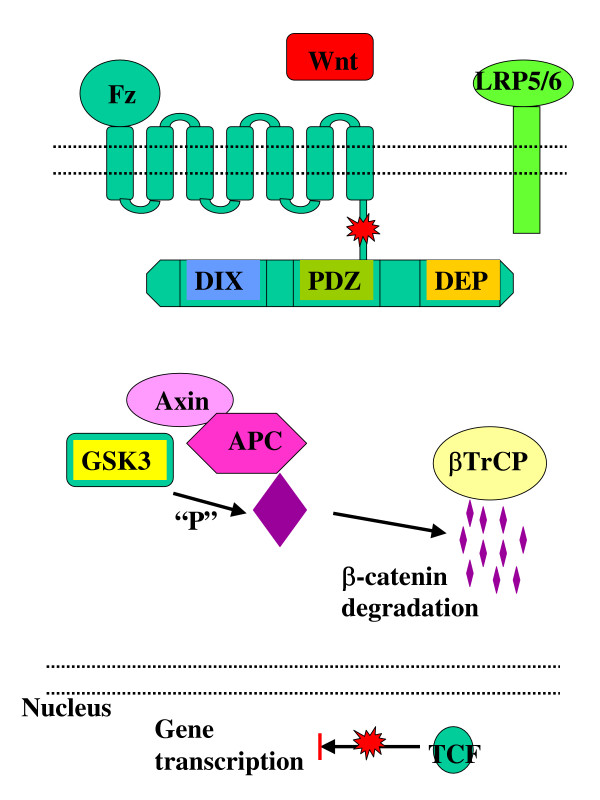
Inhibition of canonical Wnt signalling pathway in the absence of Wnt signals

**Figure 2:**
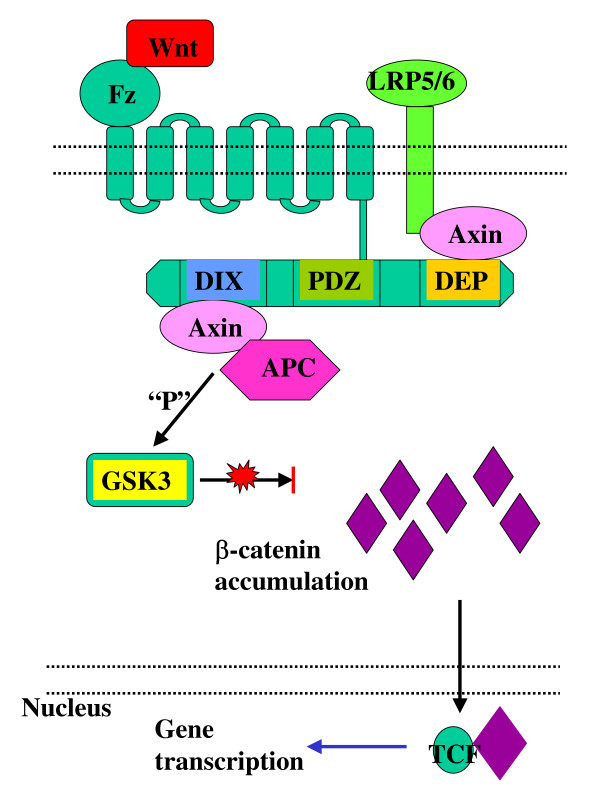
Activation of canonical Wnt signalling pathway in the presence of Wnt signals.

The canonical or β-catenin/Tcf dependent Wnt pathway was discovered first, studied most and as a result reviewed frequently [5,6]. Briefly, in the absence of Wnt signalling, glycogen synthase kinase (GSK-3) is active and phosphorylates β-catenin in the scaffolding protein complex of adenomatous polyposis coli (APC) and axin [7,8]. The phosporylated β-catenin is targeted for ubiquitination and 26S proteasome-mediated degradation, thereby decreasing the cytosolic level of β-catenin [9,10] (Figure 1). A Wnt-Fz-LRP6 complex is formed in the presence of Wnt-s that leads to the phosphorylation of three domains of Dishevelled (Dvl), which is a family of cytosolic signal transducer molecules [11]. Activation of Dvl ultimately leads to phosphorylation and consequently inhibition of GSK-3. This process is summarised in Figure 2. Inhibition of GSK3 results in stabilisation and consequently cytosolic accumulation of β-catenin (Figure 2). The accumulated β-catenin translocates to the nucleus, where it forms an active transcription complex with members of the T Cell Factor (LEF1, TCF1, TCF3, TCF4) transcription factor family [12,13] and transcription initiator p300 [14]. Successful assembly of the transcription complex leads to target gene activation. Target genes of the canonical β-catenin pathway include matrix metalloproteinases (MMP2, MMP3, MMP7, and MMP9) [15], cyclin D1 [16,17], Cox-2 [18], c-myc [19], c-jun [20], Fra-1 [20], VEGFR [21], etc. (For a recent update see Nusse's Wnt website: ).

### Non-canonical Wnt-pathways

The non-canonical Wnt pathways, the JNK/AP1 dependent, PCP and the PKC/CAMKII/NFAT dependent Ca2+ pathway (just like the canonical Wnt pathway) become activated following Wnt-Fz receptor binding [22,23]. The non-canonical pathways differ from the β-catenin pathway in their dependency on the type of G-proteins [24] they require for activation. Further downstream, Dvl is critical for signal transduction in both [25] but in contrast to canonical Wnt signalling, phosphorylation of all three domains of Dvl, is not a requirement [26]. Although the Dvl family has long been accepted as cytosol based signal transducers for the three Wnt-pathways, recent studies have revealed the ability of Dvl to translocate into the nucleus where it regulates intranuclear stability of β-catenin [27,28]. How this new function of Dvl fits into the more traditional role of the molecule awaits further investigation.

**Figure 3:**
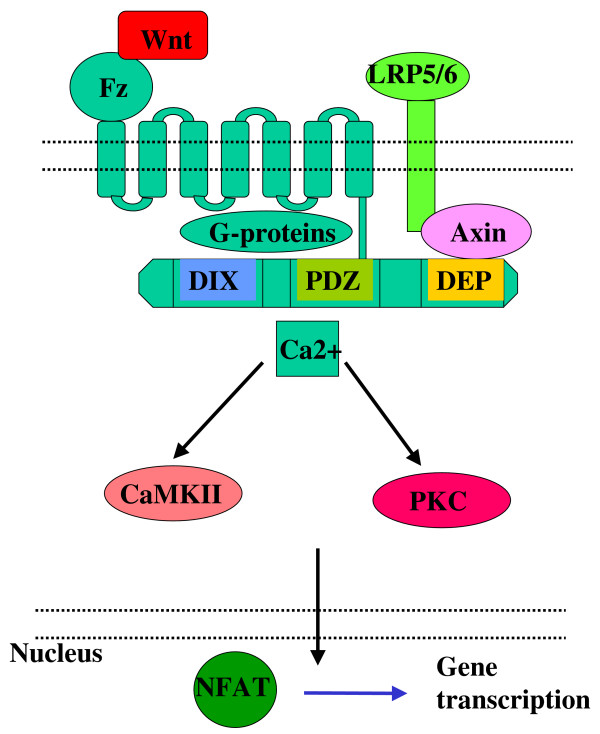
Activation of non-canonical Wnt signalling.

**Figure 4:**
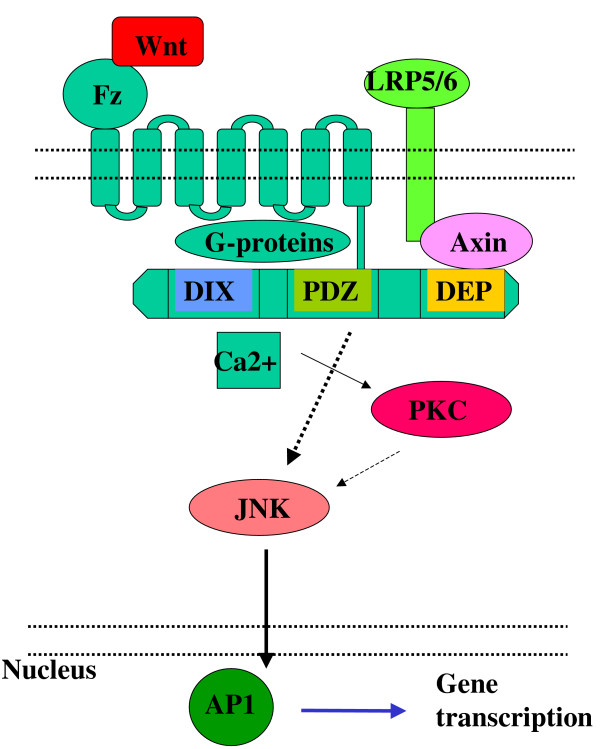
Activation of non-canonical Wnt signalling.

Nevertheless, downstream of the cytosolic Dvl, the two non-canonical Wnt pathways can activate different signalling cascades and trigger the transcription of different gene-sets, although cross-pathway activation, signal integration, and consequently gene expression modification via complex formation between NFAT and AP1 [29] can also occur. The noncanonical pathways are summarised in figure 3 and 4.

#### Ca2+ pathway

Following Dvl activation, the Ca-dependent Wnt signalling pathway activates several downstream targets including protein kinase C (PKC), Ca-Calmodulin kinase II (CaMKII), and the Ca sensitive phosphatase, calcineurin [30] before the activation of NFAT [31] occurs. NFAT is a family of transcription factors that regulate activation-induced transcription of many immunologically important genes including interleukin(IL)-2, IL-4, IFN-γ, and TNF-α [32]. Whether the genes outlined above are directly regulated by Ca2+ dependent Wnt signals has yet to be clarified. A prominent member of the non-canonical Wnt pathway activators, Wnt 5a, has recently been connected to pro-inflammatory cytokine (IL6, IL8, IL15) production [33] implicating PKC and NFkB in the process [34], although the role for both PKC and NFkB requires further conformation.

#### JNK/AP1 dependent PCP pathway

In the PCP pathway, activation of Dvl leads to JNK, and in turn to AP1 activation [35]. AP1 is not a single protein, but a complex of smaller proteins, which can form homo- and heterodimers. The main components of AP1 are cJun, JunB, JunD, cFos, FosB, Fra1, Fra2, ATF2, and CREB. The composition of the AP1 complex is a decisive factor in the selection of genes targeted for activation. Therefore regulation of the individual AP1 components is just as important as the activation or inhibition of upstream members of the pathway. cJun and Fra1, two prominent members of the AP1 complex, have been identified as target genes of the canonical Wnt signalling pathway [20], indicating yet another potential for cross-regulation between the canonical and the non-canonical Wnt pathways.

Several genes including cyclin D1 [36], MMP-3 [37], Bim [38], GMCSF [39], which are also described as Wnt target genes, are activated by AP1. Although identification of Wnt-signal dependent AP1 target genes are awaiting further investigation, recent studies have implicated both cyclin D1 and MMP-3 as direct targets of JNK-dependent Wnt signalling [40]. Intriguingly, activation of cyclin D1 gene transcription is triggered by a cFos and cJun heterodimer of the AP1 complex [41], in which cJun is a canonical β-catenin pathway target gene. It certainly raises the possibility, that regulation of cyclin D1 expression by the PCP pathway is also influenced indirectly through canonical Wnt signalling.

## Regulation of Wnt signalling

The highly complex Wnt signalling pathways are central to the regulation of a wide range of cell functions and therefore tightly controlled. An armada of secreted extracellular (DKK-s [42], sFRP-s [43,44], WIF [45], Cer [46]) and intracellular, both cytosolic (ICAT [47-49], Nkd [50]) and nuclear (Sox17 [51]), signal modulators make Wnt signalling difficult to decipher. Further to individual inhibitors, there is also cross-talk amongst different Wnt signaling pathways. The non-canonical pathways, for example, can also act as regulators of canonical Wnt signalling, often by influencing the phosphorylation and therefore activation state of GSK (one of the main enzymes of the canonical Wnt pathway) [52,53].

**Figure 5:**
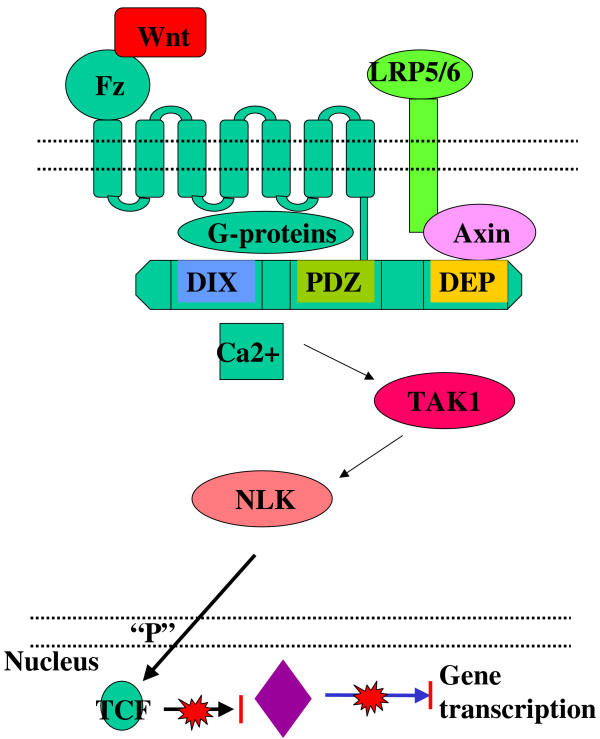
Inhibition of Wnt signalling by a Fz-dependent pathway.

Furthermore, inhibitory Fz pathways have also been described. Fz1 [54,55] inhibits Wnt signal transduction via a G-protein dependent manner. The other inhibitory Fz, Fz6, [56], inhibits Wnt dependent gene transcription by activating a Ca dependent signalling cascade involving TAK1 and Nemo-Like Kinase (NLK) [57,58], and ends with the phosphorylation of TCF family members. The resulting structural changes in TCF-s inhibit β-catenin TCF binding and consequently activation of gene transcription [57] (Figure 5).

### Wnt signalling in the developing lung

Modulation of Wnt expression in embryonic and adult mouse lung suggests that Wnt pathways are important for cell fate decisions and differentiation of lung cell types. The involvement of canonical Wnt signalling in lung development has been proven by several ways. A TCF promoter-LacZ based reporter system has shown, that canonical Wnt signalling is active throughout lung development in mouse embryos [59]. β-catenin, a central molecule of canonical Wnt signalling, has been shown to localize in the cytoplasm, and often also the nucleus of the undifferentiated primordial epithelium (PE), differentiating alveolar epithelium (AE), and adjacent mesenchyme [60]. Using a conditional knockout system for β-catenin in mice has also revealed that β-catenin dependent signalling is central to the formation of the peripheral airways of the lungs, responsible for conducting gas exchange, but is dispensable for the formation of the proximal airways [61]. Constitutive activation of the canonical Wnt pathway using a β-catenin-Lef1 fusion protein, produced a similar effect [59]. Although proximal airways developed, the lung was reduced in size and lacked alveoli [59].

Recent studies have related particular Wnt production to specific lung cell types. Wnt2 [62] for example has been mapped predominantly to the mesenchyme, Wnt11 to both epithelium and mesenchyme [63], while Wnt7b was exclusively expressed in the lung epithelium [64]. Additional studies have revealed that Wnt7b promoter activity is regulated by a homeodomain transcription factor, TTF1, which is essential to the differentiation of lung epithelium, being especially important for the highly specialised Type II alveolar epithelial cells [65]. Since the TTF1 null mice have a lethal lung phenotype with increased epithelial and mesenchymal proliferation, which at the neonatal stage contains abundant mesenchyme and no functional alveoli [65], it is likely that the lack of functional alveoli is a result of dysregulated Wnt7b signalling [64].

Apart from β-catenin and Wnt-s, mRNA of Fz-1, -2 and -7 and several intracellular signalling molecules including Tcf-1, -3, -4, Lef1, and secreted Fz related proteins (sFrp-1, -2 and -4) have been found to be expressed in the developing lung [60] in specific, spatio-temporal patterns [60]. Wnt signalling has also been reported to be important in the regulation of spatial and distal branching of the lung [61].

While the importance of canonical Wnt signalling in lung development is well established, the role of non-canonical Wnt signalling is less clear. Wnt5a knock-out studies have shown, however, that non-canonical Wnt signalling is also important. In Wnt5a-/- animals the lung is morphologically smaller than in the wild type [66] and has thickened mesenchyme. Furthermore, alveolar development is delayed, although not prevented [66]. Lungs of Wnt5a knock-out animals also have increased expression of FGF10 and Shh [66,67] suggesting that the morphological changes might be related to dysregulation of other signalling pathways modulated by Wnt signalling (see below for further details).

### Wnt-s in adult lung

Primary lung tissue and cell lines, derived from adult lung tissue, express a wide range of Wnt-s including Wnt-3, -4, -5a, -7a, -7b, -10b, and -11 [68], as well as Fz-3, -6 and -7 [68], Dvl [69], and Dkk [70]. Since, generally, Wnt signalling retains cells in a low differentiation state, the role of Wnt signalling in adult tissue may not be immediately clear. If we assume that the maintenance of adult organs is stem cell dependent and that stem cells rely on β-catenin and Tcf/Lef signalling to be maintained in the required low differentiation level, the role of Wnt signals in adult tissue becomes understandable. Stem cell niches in proximal and distal airways exist [71,72], similarly to intestine, hair follicle and dermis, and would need Wnt signalling to be able to fulfill their role in maintenance of adult lung structure.

### Wnt in lung carcinoma

While lung cancer is one of the leading causes of cancer deaths worldwide [73,74] data regarding the role of Wnt pathways in human lung cancer is still limited. The most studied pathway mutations in cancer are the inherited and sporadic mutations in the tumour suppressor adenomatous polyposis coli (APC) and β-catenin. Since APC is part of the degradation scaffold for β-catenin, mutations of APC can result in reduced degradation and increased nuclear accumulation of β-catenin leading to activation of target genes such as oncogenes cyclin D1 and c-myc [75]. Degradation resistant β-catenin has similar effect on target gene activation [59]. Although increased levels of β-catenin have been reported in different types of lung cancers [76,77], mutations of APC [78] and β-catenin [79,80] are rare in lung cancers. However, proof of dysregulation of specific Wnt molecules leading to oncogenic signalling has emerged. While frequent loss of Wnt7a mRNA was demonstrated in some studies in lung cancer cell lines and primary tumours [81], elevated levels of Wnt1 [82] and Wnt2 [83] have been reported in non small cell lung cancer. Decreased levels of Wnt7a indicates that Wnt7a may function as a tumour suppressor in lung cancer. In support this concept, non-small-cell lung cancer cells transformed with Wnt7a showed inhibition of anchorage independent growth [68]. Although member of the canonical group, Wnt7a inhibits proliferation and induces differentiation via the JNK/AP1 dependent PCP signalling pathway [68]. The role of non-canonical Wnt signalling in the development of lung cancer remains controversial despite recent findings. Although the non-canonical pathway activator Wnt5a is an important regulator of lung development, and generally is an inhibitor of canonical Wnt signalling, elevated levels of Wnt5a in lung metastases of human sarcoma [84] has been reported and thus questions the role of non-canonical Wnt signalling as a general inhibitor of lung cancer. In metastatic stage of any tumours including human lung carcinomas, epithelial-mesenchymal transformation (EMT) is typical [85] and generally linked to increased β-catenin dependent signalling [86]. As β-catenin mutations in lung cancers are relatively rare [79,80,87], another possible mechanism might be at place which regulates EMT and consequently tumour metastasis in the lung. Certainly, non-canonical Wnt5a the very molecule which has recently been reported to regulate fibroblast growth factor (FGF) 10 and sonic hedgehog (Shh) expression [67] has been found elevated in lung metastases [84]. Both FGF-s and the hedgehog family are well-known modulators of epithelial-mesenchymal interactions [88] and epithelial-mesenchymal transformations (EMT) [89-91]. Dysregulation of FGF and Shh signalling certainly raises the possibility that Wnt5a and perhaps non-canonical Wnt signalling in general, is indirect regulator of lung tumour metastasis.

Lung developmental studies have also provided support for the involvement of canonical Wnt signalling in lung cancer. Constitutive activation of the canonical pathway in the developing lung resulted in a non-differentiated lung phenotype resembling cancer [59]. Target genes of the canonical and PCP Wnt pathways include matrix metalloproteinases, which are essential for tissue remodelling and are elevated in invasive cancer [92,93], thus providing additional evidence for the involvement of Wnt signalling in lung cancer.

Overexpression of Dvl, a positive regulator of Wnt signalling pathways has been reported in 75% of non-small-cell-lung-cancer samples compared with autologous matched normal tissue [94]. Downregulation of Wnt pathway antagonists like Dkk3 [70], WIF [95,96] and sFRP [97] have also been reported in various types of lung cancers providing further evidence of the role of this complex pathway.

### Wnt in lung inflammation

To date there is no direct evidence for the involvement of Wnt signalling in inflammation of the central airways. However, based on the general features of inflammatory diseases and evidence for Wnt regulated signalling in inflammation in the joint [34], we have addressed the potential involvement of Wnt signalling in inflammatory diseases of the lung.

Increased levels of pro-inflammatory and inflammatory cytokines such as IL1, IL6, IL8, and IL15, monocyte chemotactic protein-1 (MCP-1), TNFα and intercellular adhesion molecule-1 (ICAM-1) are general features of inflammation. The elevated expression of ICAM in the epithelium is important in leukocyte recruitment, adhesion and retention [98], while IL8 secreted by the bronchial epithelium [99], is thought to be central to the attraction of neutrophils. Neutrophils together with macrophages contribute to the pathogenesis of inflammatory tissue injury by reactive oxygen metabolites and proteinase release. Increased levels of tissue matrix metalloproteinases (MMP-s) are a feature of inflammatory conditions and may contribute to the overall evolution of the inflammation-induced tissue destruction. Several pulmonary cells including resident alveolar macrophages, neutrophils, parenchymal cells (including interstitial fibroblasts), type II epithelial cells and vascular endothelial cells are capable of elaborating MMPs [100], and numerous MMP-s, including MMP3 and MMP9, have been considered to have important pro-inflammatory roles in acute lung inflammation [101]. Activation of MMP gene transcription has been attributed to both pro-inflammatory cytokines [102,103] and canonical Wnt signalling [15], but it is still not clear whether they act in competition or in close connection to regulate the transcription of MMP genes. Certainly, the canonical pathway activator Wnt-1 has been linked to stimulation of pro-MMP3 transcription [104], which is implicated in lung inflammation [105]. Understanding of signalling pathway interaction is thus of importance in the study of pathogenic processes and hence disease modulation.

Studies of rheumatoid arthritis have accumulated evidence that Wnt5a-Fz5 mediated signalling can contribute significantly to the production of pro-inflammatory cytokines (IL6, IL8, IL15) [33] and that overexpression of Wnt5a leads to increased pro-inflammatory cytokine levels. Furthermore, dominant negative and antisense Wnt5a and anti-Fz-5 antibody block Wnt5-Fz5 signalling leading to decreased cytokine production [33].

Additionally, the inflammatory cytokine inducing Wnt5a has also been implicated in the down-regulation of Shh levels in the lung [67]. Elevated Shh signalling is well established in the regulation of inflammatory and fibrotic processes of the gut and lung [91]. This suggests a role for Wnt5a but further investigation would be necessary to clarify this in the central airways- in pulmonary inflammation.

### Wnt in lung fibrosis

Lung diseases resulting in tissue damage activate a defence mechanism to repair the lesions. Tissue damage can result from several acute and chronic stimuli including inflammation caused by infections, autoimmune reactions (asthma, allergic alveolitis), and drugs and toxins (bleomycin, asbestos) or mechanical injury (surgery, and irradiation). Any tissue repair involves coordinated cellular infiltration together with extracellular matrix deposition and where appropriate, re-epitheliasation. In the first regenerative step, injured cells are replaced by cells of the same type, then normal parenchyma is replaced by connective tissue leading to fibrosis. Usually both steps are required for healing, however, when the fibrotic step becomes uncontrolled and pathogenic, the process can lead to organ failure and death. The interstitial lung disease (ILD) includes a wide range of disorders in which pulmonary inflammation and fibrosis are the final common pathway.

Generally, any activated state of tissue repair requires the stimulation of signalling pathways involved in proliferation, cell migration and differentiation. It is therefore understandable that the fibrotic process is influenced by a combination of growth factors (such as TGFβ, FGF), and cell adhesion molecules (such as integrins). Modulation of growth factor expression, loss of E-cadherin and activation of β-catenin dependent gene transcription leads to epithelial-mesenchymal transition (EMT) which is also an important feature of the fibrotic process. Direct involvement of canonical Wnt signalling in EMT has been confirmed in studies using Wnt1 and Lef-1 overexpression [106]. Furthermore, during cellular migration, which is an important factor in tissue repair, proteolytic degradation of the extracellular matrix is necessary to enable fibroblasts to migrate through the extracellular matrix to the site of the lesion. Proteolytic degradation of the extracellular matrix requires plasminogen and matrix metalloproteinases [107,108]. Gene transcription of MMP-s is regulated by Wnt signalling of both canonical and non-canonical pathways. Metalloproteinase matrilysin (MMP7), a target gene of the canonical Wnt signalling pathway [109], has recently been identified as a key regulator of pulmonary fibrosis [110,111]. In many cases of idiopathic pulmonary fibrosis, the levels of nuclear β-catenin are elevated [112], as are the levels of β-catenin target genes, cyclin D1 and MMP-s [112].

As Wnt-s have also been implicated in the modulation of proliferation and differentiation of many lung cells [59,60,66], the role of Wnt signalling in regulating cell proliferation and differentiation during idiopathic pulmonary fibrosis, is likely to be central rather than a consequence of the disease.

In summary, Wnt signalling may also be central to all causes of pulmonary fibrosis and requires further evaluation.

## Interaction of Wnt pathways with FGF, TGFβ /BMP/Smad pathways

Although detailed discussion of interactions of Wnt with other signalling pathways is not the aim of the present review, it is still important to highlight some regulatory interactions, which might also play a role in development and control of pulmonary diseases. Certainly, the non-canonical pathway activator Wnt5a has been implicated in the regulation of several signalling pathways. In Wn5a-/- knockout animals there is increased FGF10 and BMP4 expression [66] suggesting a key role of Wnt5a in the regulation of both factors. Since FGF10 stimulates proliferation and branching in the developing lung and also induces delayed distal epithelial BMP4 expression, which eventually inhibits lung bud outgrowth [113], Wnt5a appears to be a key regulator of cellular proliferation in the lung.

The effect of Wnt-s as signal modulators of other signalling pathways has also been demonstrated. For example, the canonical Wnt pathway inhibitor, ICAT [47], regulates the expression of the BMP pathway inhibitor, BAMBI (BMP and activin membrane-bound inhibitor) [114]. Since ICAT functions by blocking binding sites of TCF-s and p300 on the armadillo domains of β-catenin [47] and therefore inhibiting β-catenin dependent gene transcription, this suggests that BAMBI is not only directly controlled by BMP4 [115] but also by canonical Wnt signalling.

Moreover, both the TGFβ and BMP pathways require Smad-s (reviewed in [116]) for signal transduction but Smad-dependent gene transcription can also be modulated by β-catenin [117,118], binding to Smad-nuclear complexes. A role for the Smad-system activator TGFβ 1 in pulmonary fibrogenesis has recently been confirmed [119]. It was shown that TGFβ 1 has a direct role in regulating EMT by promoting alveolar epithelial cell transition to form mesenchymal cells with a myofibroblast-like phenotype. As both TGFβ and β-catenin signalling induces EMT, a Wnt/TGF signal interaction became evident once again emphasising the need for further studies to define details of signal transduction and pathway coordination to fully understand the underlying processes of EMT.

Since FGF, Shh, TGFβ, and BMP signalling pathways are all important in tissue repair, fibrosis and cancer invasion, it appears, that Wnt signalling can modulate disease progression both directly and indirectly by activating gene transcription and modulating and cross-regulating signalling pathways.

## Summary

The involvement of Wnt signalling in lung development, maintenance, cancer, and repair (including idiopathic pulmonary fibrosis) is supported by evidence, while based on indirect evidence a role for Wnt signalling in inflammatory lung diseases can also be postulated. Certainly, better understanding of Wnt signalling in the lung is likely to be important and provide information central to new treatment approaches for a wide variety of lung diseases.
